# Differential utilization of binding loop flexibility in T cell receptor ligand selection and cross-reactivity

**DOI:** 10.1038/srep25070

**Published:** 2016-04-27

**Authors:** Cory M. Ayres, Daniel R. Scott, Steven A. Corcelli, Brian M. Baker

**Affiliations:** 1 Department of Chemistry & Biochemistry, University of Notre Dame, Notre Dame, IN 46556 USA; 2 Harper Cancer Research Institute, University of Notre Dame, Notre Dame, IN 46556 USA

## Abstract

Complementarity determining region (CDR) loop flexibility has been suggested to play an important role in the selection and binding of ligands by T cell receptors (TCRs) of the cellular immune system. However, questions remain regarding the role of loop motion in TCR binding, and crystallographic structures have raised questions about the extent to which generalizations can be made. Here we studied the flexibility of two structurally well characterized αβ TCRs, A6 and DMF5. We found that the two receptors utilize loop motion very differently in ligand binding and cross-reactivity. While the loops of A6 move rapidly in an uncorrelated fashion, those of DMF5 are substantially less mobile. Accordingly, the mechanisms of binding and cross-reactivity are very different between the two TCRs: whereas A6 relies on conformational selection to select and bind different ligands, DMF5 uses a more rigid, permissive architecture with greater reliance on slower motions or induced-fit. In addition to binding site flexibility, we also explored whether ligand-binding resulted in common dynamical changes in A6 and DMF5 that could contribute to TCR triggering. Although binding-linked motional changes propagated throughout both receptors, no common features were observed, suggesting that changes in nanosecond-level TCR structural dynamics do not contribute to T cell signaling.

T cell cross-reactivity between different peptide antigens bound and presented by major histocompatibility complex molecules (peptide/MHCs) is central to cellular immunity, permitting a fixed size T cell repertoire to respond to a substantially larger universe of potential antigens^1^. By some estimates, a single T cell can recognize as many as 10^6^ different peptide/MHCs^2^. T cell cross-reactivity is facilitated in part by the structural versatility of the T cell receptor (TCR) (reviewed in ref. 3). In many cases, it has been shown that conformational changes within TCR complementarity determining region (CDR) loops allow the receptor to adjust to different ligands (e.g., refs 4, 5, 6, 7). A role for conformational changes in TCR binding was implied by early thermodynamic measurements^8^,^9^, and incorporated into mechanisms for how TCRs might “scan” for compatible MHC-presented peptides on antigen presenting cells via induced-fit-type mechanisms^10^.

As additional structural data has emerged, it has become clear that extensive conformational changes are not always necessary for TCR binding and cross-reactivity^11^. Binding of the same TCR to different ligands can occur by rigid body changes in how a TCR sits over a peptide/MHC ligand^12^,^13^,^14^, via adaptive changes in the ligand^15^,^16^, or by simply accommodating different ligands via permissive architectures^13^,^17^. Additionally, because fewer structures of unligated TCRs are available compared to those of TCR-peptide/MHC complexes, the extent of conformational changes occurring upon binding is often unknown.

Often lacking in discussions about the roles of TCR conformational changes in ligand binding is knowledge of the underlying TCR conformational dynamics, as these cannot be assessed by crystallographic structures alone. Insight into motion is important for understanding mechanisms of ligand binding, selection, and cross-reactivity, and can influence efforts in TCR engineering. For example, the αβ TCR 2C alters its conformation upon binding pMHC^5^,^14^, and these motions are reflected in the properties of the free TCR^18^. Similar results have been shown for the A6 TCR: by combining crystallography with molecular dynamics simulations and experimental measurements of motion and binding, we showed that these conformational differences are facilitated by conformational changes occurring on the nanosecond timescale^19^,^20^. Indeed, the CDR3β loop of the unligated A6 TCR was found to sample all of its crystallographically observed conformations, promoting a binding mechanism better described by a conformational selection rather than induced-fit mechanism^21^. The relevance of this data was further demonstrated by the rational design of high affinity A6 TCR variants through the introduction of mutations that limited CDR3β loop motion^22^. For 2C, although it undergoes a reduction in dynamics upon binding, complementary receptor/ligand motion within the interface continues within the complex, permitting the retention of key interactions across the interface^18^, foreshadowing the discovery of how TCR-peptide “hot spots” facilitate cross-reactivity^23^.

To broaden our understanding of the motional properties of TCRs and how these influence ligand binding and selection, here we compared the dynamics of the αβ TCR A6 with those of another structurally well-characterized TCR, DMF5^13^. We used molecular dynamics simulations validated with measurements of fluorescence anisotropy. A6 (*TRAV12-2; TRBV6-5*) recognizes the HTLV-1 Tax 11–19 peptide (sequence LLFGYPVYV), numerous Tax variants, and related peptides all presented by the class I MHC protein HLA-A*0201 (HLA-A2). DMF5 (*TRAV12-2; TRBV6*–*4*) recognizes the melanoma-associated MART-1 26–35 decamer (EAAGIGILTV), 27–35 nonamer (AAGIGILTV), and variants of both, also presented by HLA-A2. Structurally, A6 and DMF5 might be considered opposite ends of a spectrum: A6 displays considerable conformational differences when free and or bound to different ligands as discussed above, whereas DMF5 binds different ligands identically, with smaller conformational differences between the bound and free states. Although they show different structural properties, A6 and DMF5 share the same Vα gene segment. Therefore, in addition to illuminating the motional properties of two structurally divergent TCRs, these receptors provide the opportunity to examine if and how motions of common germline loops are influenced by dissimilar neighbors. In addition to the CDR loop motion in the free TCRs, we also examined motional changes that occur upon ligand binding and whether changes that might contribute to the mechanism of TCR triggering could be identified^24^.

Overall, our results illustrate two different strategies used by TCRs to bind different peptide/MHC ligands. Consistent with our prior work, we found the binding site of the A6 TCR to be highly dynamic, incorporating substantial uncorrelated motion occurring on the nanosecond timescale. The binding site of DMF5 on the other hand, was more rigid, incorporating smaller, more correlated motions. The data thus reflected different mechanisms of ligand selection and cross-reactivity. Motion for both TCRs was altered upon binding, yet consistent with the different cross-reactivity mechanisms, the interface formed by DMF5 TCR remained more fluid than the interface formed by A6.

Lastly, we were unable to identify dynamic changes upon binding that might be associated with TCR triggering. Altogether, our results expand our understanding of how motion impacts TCR binding, ligand selection, and cross-reactivity, and provide a point for further studies of how binding-linked changes might contribute to T cell signaling. They indicate that simplifying generalizations about the role of flexibility in TCR functional properties cannot easily be made, and provide data for how motion may be accounted for in efforts to manipulate TCR binding.

## Results

### The CDR loops of the DMF5 T cell receptor are less mobile than those of A6

**Figure 1:**
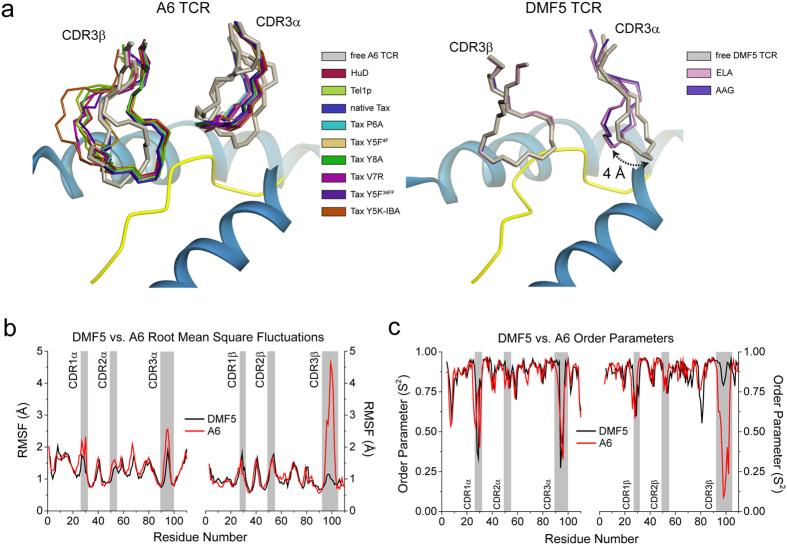
Differential dynamics of the A6 and DMF5 CDR binding loops. (**a**) Comparison of the conformations of the hypervariable CDR3α and CDR3β loops of A6 (left) and DMF5 (right) in bound and free crystal structures. The loops of the A6 TCR show considerable structural variation when structures are compared, whereas those of the DMF5 TCR are in the same conformation when bound to the MART-1 26-35 decamer or 27–35 nonamer, with a small 4 Å shift in the position of the CDR3α loop between free and bound (left hand image adapted from ref. 20). (**b**) RMS fluctuations for the A6 and DMF5 variable domains computed from 500 ns of MD simulation of the free A6 and DMF5 TCRs. The shaded boxes indicate the positions and values of the various CDR loops. (**C**) As in panel B, but for order parameters.

The A6 TCR exemplifies how a T cell receptor can utilize loop flexibility to recognize and cross-react between different ligands^20^. However, structural data for the DMF5 TCR suggests that significant flexibility is not always required for either TCR binding or cross-reactivity^13^. The different behaviors exemplified by A6 and DMF5 are illustrated structurally in Fig. 1a. The conformation of the A6 CDR3β loop varies dramatically with different structures (including free and bound), while CDR3α twists and reorganizes into a common conformation upon binding. In the DMF5 TCR, the conformation of the CDR3β loop is invariant whether free or bound to different ligands, and CDR3α (and to a lesser extent, CDR1α) undergoes a comparatively simple hinge movement between its free and bound states. While there are more structures available for A6 than DMF5, the stark differences between the two, particularly when comparing the free and bound states, suggests potential differences in the two TCRs’ motional properties.

We previously demonstrated that the conformational differences seen for the A6 TCR were related to rapid motions occurring on the nanosecond timescale in the free TCR^20^. To compare the nanosecond-scale flexibility of DMF5 with A6, we examined the free DMF5 TCR with a 500 ns, multi-trajectory molecular dynamics simulation in explicit solvent. To ensure an appropriate comparison, we also performed a new, identical simulation of free A6. In both cases we began with available structures of the unligated TCRs^13^,^20^. For each simulation we superimposed the α carbons of the entire protein in each picosecond frame and computed root mean square (RMS) fluctuations of the α carbons of each CDR loop. This analysis revealed marked differences in the amplitudes of motion of the CDR loops of the two TCRs, most profoundly in CDR3β (Fig. 1b). The CDR3α and CDR3β loops of the A6 TCR were the most mobile, with RMS fluctuations at the apex close to 3 Å for CDR3α and 5 Å for CDR3β. In contrast, the CDR3α and CDR3β loops of DMF5 were more rigid, with maximum RMS fluctuations near 2 Å and 1 Å, respectively. The fluctuations of the DMF5 CDR3β loop were the lowest of all, including the germline loops of both receptors. The differences between the A6 and DMF5 hypervariable loops could be related to length: as shown in Table 1, the CDR3α and CDR3β loops in A6 are longer than in DMF5 (11 and 14 amino acids for CDR3α and CDR3β in A6, vs. 10 and 11 for CDR3α and CDR3β in DMF5).

There were less pronounced differences in the fluctuations of the A6 and DMF5 germline loops. However, the mobility of the A6 CDR1α and CDR2α loops were slightly enhanced relative to those of DMF5 (Fig. 1b). This difference is notable as DMF5 and A6 share the same *TRAV12-2* α chain, and the DMF5 and A6 CDR1α/CDR2α loops are therefore identical in length and composition (Table 1). As discussed below, the differences in the motional properties of these otherwise identical loops may arise from different lateral interactions with the hypervariable loops.

We also computed order parameters for each backbone Cα–Cβ bond (Cα-H for glycine) providing complementary information on motional timescale (Fig. 1c). In general, there was close agreement between the order parameter and fluctuation data, particularly in the considerable differences between the A6 and DMF5 CDR3β loops.

For the A6 TCR, we previously performed a comprehensive experimental validation of our molecular simulation methodology using fluorescence anisotropy^25^. To ask if the simulation with the DMF5 protein performed similarly well, we fluorescently labeled positions in the DMF5 CDR1α loop and the CDR3β loop. Arg27α and Phe100β were each mutated to cysteine and labeled with fluorescein-5-maleimide. These sites were chosen as the original side chains are solvent exposed and do not interact with neighboring groups. Further, the simulations indicate different levels of backbone mobility at these sites, with the position in the CDR3β loop more rigid than the position in the CDR1α loop (Fig. 1b,c). Steady state anisotropy measurements were in agreement with these results: the value for protein labeled in CDR3β was 0.23, whereas the value for protein labeled in CDR1α was a more flexible 0.15 (the theoretical maximum for fluorescein, reflecting a molecule that is immobile over the fluorescence lifetime, is 0.4) (Fig. S1A). However, as we demonstrated previously^25^, incorporation of a fluorescent label can alter the motional properties of TCR loops, such that measurements on labeled proteins may not reflect the behavior of unlabeled proteins. We thus performed two separate 100 ns molecular dynamics simulations of the DMF5 TCR incorporating the cysteine and attached fluorescent label at positions 27α or 100β. The RMS fluctuations and order parameters for the labeled sites were close to the values for the labeled protein and did not significantly alter values for neighboring amino acids (Fig. S1B), confirming that, as with the A6 TCR, the simulation methodology accurately reports on the nanosecond motional properties of the DMF5 receptor.

### Incomplete conformational sampling suggests induced-fit behavior for the DMF5 TCR

**Figure 2:**
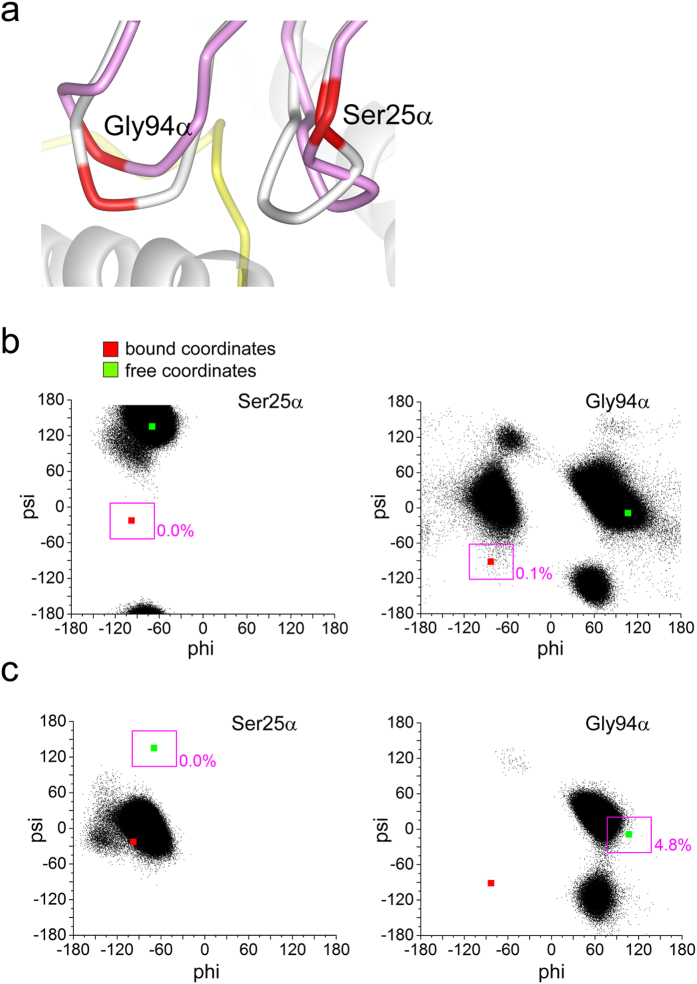
Slower movement of DMF5 CDR loops suggest induced-fit behavior for a binding-linked conformational change. (**a**) Comparison of the structures of the DMF5 CDR3α and CDR1α loop in their bound (purple) and free (grey) conformations, with Ser25α and Gly94α highlighted in red. The peptide and part of the MHC α1/α2 helices are shown in yellow and grey for orientation. (**b**) Phi/psi angle distributions from 500 ns of MD simulation of free DMF5 for Ser25α (left) and Gly94α (right). Green squares show the angles in the crystallographic structure of the free TCR. Red squares show the angles in the crystallographic structures of the bound TCR. Boxes indicate ±30° from the bound-state angles, with percentage of sampling within the box indicated. The bound conformation is not sampled for Ser25α, and only to 0.1% for Gly94α. (**c**) Phi/psi distribution from 500 ns for Ser25α (left) and Gly94α (right) of MD simulation of free DMF5, but beginning with the coordinates of the bound-state. Figures are notated as above. For Ser25α the loop does not return to its free-state conformation over the course of the simulation, indicative of a high-energy barrier between the bound and free conformations. For Gly94α the loop moves close to but does not fully arrive at the conformation seen in the free TCR.

Although the CDR loops of the DMF5 TCR undergo smaller fluctuations than those of A6, backbone conformational changes are still required for the DMF5 TCR to bind. These changes are localized to the CDR3α and CDR1α loops, which shift in order to “open” a groove that accommodates the N-terminal end of the peptide (Figs 1a and 2a). However, the open conformation was not sampled over the course of the simulation of the free TCR. This is most easily demonstrated by examining ϕ/ψ angle distributions: residues in the DMF5 CDR1α and CDR3α loops did not sample (or in some cases, only rarely sampled) configurations seen in the bound state, instead sampling the free and a small subset of other unrelated conformations (Fig. 2b).

The results for the DMF5 CDR1α and CDR3α loops suggest the bound and free conformations are separated by high energy barriers. To verify this, we performed a 500 ns simulation of the free DMF5 TCR beginning with the coordinates of the receptor bound to the MART-1 26–35 ELAGIGILTV decamer/HLA–A2 ligand (i.e., starting with the loops in their bound state conformation). In this simulation, CDR3α moved close to but did not fully adopt the conformation seen in the unbound TCR, and CDR1α remained fully in its bound-state conformation (Fig. 2c). These results for the DMF5 TCR differ substantially from those seen for the A6 TCR, in which the loops that adapt to ligand rapidly sampled all known crystallographic conformations^20^. Thus, as opposed to A6, the data suggest that backbone conformational changes required for DMF5 to bind reflect a greater contribution of slow or induced-fit-type motions.

### The binding site of A6 is dynamic and motionally independent whereas DMF5 is rigid and correlated

**Figure 3:**
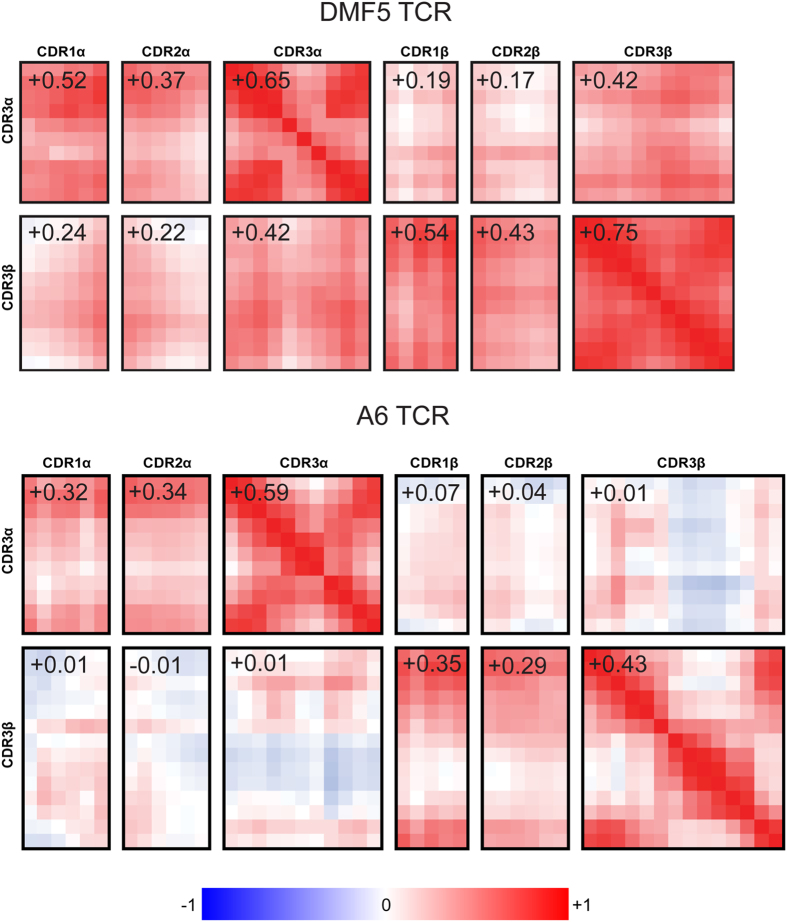
The CDR loop motions of DMF5 (top) are positively correlated, whereas those of A6 (bottom) are largely uncorrelated. Each large rectangle indicates a cross-correlation matrix for pairs of the CDR loops, with the individual squares indicating motional correlations between the α carbons of each amino acid of each loop. Numbers are the average correlation value for the data in each rectangle, yielding the average correlations between the CDR loops. The color scale from −1 (blue; full anti-correlation) to +1 (red; full correlation) is indicated at the bottom.

To examine the motional relationships across the two TCR binding sites, we next examined how motion was correlated between the various CDR loops of the A6 and DMF5 TCRs, computing dynamic cross-correlation matrices for both simulations. The most marked differences between the receptors was in the behavior of the hypervariable loops. In the DMF5 TCR the motions of the CDR3α and CDR3β loops were positively correlated: on the scale from −1 (fully anti-correlated) to +1 (fully correlated), the average correlation coefficient for backbone α carbons of the CDR3α/CDR3β loops was +0.42 (Fig. 3a). The DMF5 hypervariable loops were also positively correlated with the α and β chain germline loops, with average correlation coefficients for pairs of different loops ranging from +0.17 to +0.54.

In contrast with DMF5, the more mobile CDR3α and CDR3β loops of A6 moved independently of each other, with an average correlation coefficient near zero (+0.01) (Fig. 3b). Also unlike DMF5, there was no correlation across the A6 α and β chains, as CDR3β motions were not correlated with those of CDR1α or CDR2α, nor were CDR3α motions correlated with those of CDR1β or CDR2β. The picture that emerges thus far is that in its free state, the A6 TCR possesses a binding site with a highly dynamic yet motionally independent center, surrounded by a more rigid periphery. In contrast, the binding site of the DMF5 TCR is in general more rigid, incorporating small correlated motions between all loops.

### Structural correlations with differential motion in A6 and DMF5

We next asked whether the dynamical differences between the two TCRs could be predicted from features apparent in the crystallographic structures of the unligated TCRs. At the simplest level, the quality of electron density correlated with the differences in dynamics of the A6 and DMF5 CDR3 loops. In the structure of the A6 TCR, electron density for portions of CDR3α and CDR3β was weak or even missing^20^, whereas clear density was present in DMF5^13^. As the CDR3α and CDR3β loops in A6 are the most dynamic, electron density is thus a qualitative indicator of the differential motional properties between the two TCRs.

**Figure 4:**
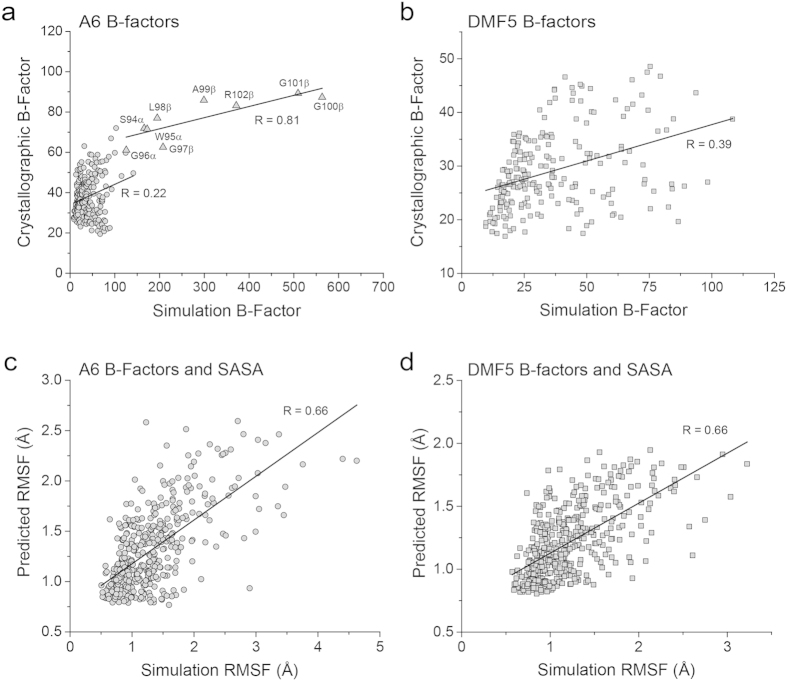
Structural correlations with motional amplitudes. (**a**) Crystallographic vs. simulation B-factors for CDR loop α carbons in the free A6 TCR simulation. The observed vs. computed values for the apex of the highly mobile CDR3β loop (triangles) are well-correlated (R = 0.81). The values for the remainder of the CDR loops, however, are poorly correlated (R = 0.22). (**b**) As in panel A, but for the free DMF5 TCR. The values are poorly correlated, with R = 0.39. (**c**) Combining α carbon crystallographic B-factors with residue-specific relative solvent accessible surface areas led to a correlation between predicted and simulation RMSF values for the residues of the A6 CDR loops of 0.66. Weights for the linear terms are 0.012 for B-factors, 0.009 for surface area, and 0.5 for the intercept. (**d**) As in panel C, but for the DMF5 TCR. Weights for the linear terms are 0.014 for B-factors, 0.0075 for relative SASA, and 0.58 for the intercept.

In looking for more quantitative correlations, crystallographic B-factors clearly identified the greater mobility of the apexes of the A6 CDR3α and CDR3β loops: as shown in Fig. 4a, the B-factors for the α carbons of the apexes of the A6 hypervariable loops were well-correlated with those computed from RMS fluctuations, with a correlation coefficient (R) of 0.81. However, the correlation was weaker for the other loops of A6 (R = 0.22), as well as for the loops of the less mobile DMF5 TCR (Fig. 4b; R = 0.39).

We next asked if structural features in the crystallographic coordinates were useful in predicting the dynamic differences between A6 and DMF5. The presence or absence of interloop hydrogen bonds correlated poorly: DMF5 has six interloop hydrogen bonds, compared to five with A6. While the more dynamic A6 TCR has one fewer hydrogen bond, in both cases all hydrogen bonds are to the CDR3β loop, whose dynamics differ most significantly between the two TCRs (Fig. 1). Loop sequence was also not a strong indicator of dynamics: the apex of the highly flexible CDR3β loop of A6 has a two-glycine motif (sequence AGGR; Table 1), yet the apex of the considerably more rigid CDR3α loop of DMF5 has a three-glycine motif (sequence FGGGK).

In a simple examination of the structures, the interfacial region of the A6 TCR appeared more “open” than that of DMF5. We thus asked if residue-specific solvent accessible surface areas (SASA) or atomic packing density throughout the two proteins correlated with motional properties. We observed similar correlations of both with RMS fluctuations (R = 0.58). Combining relative SASA with crystallographic B-factors via multi-linear regression yielded models for both TCRs that allowed for better estimation of fluctuations: linear equations with parameters for B-factors and relative SASA generated correlations between predicted and measured RMSF of 0.66 for both TCRs, with similar weights for both A6 and DMF5 (Fig. 4c,d). This analysis gives some insight into how motional properties could be discerned from crystallographic structures, but clearly other factors have an influence. We note that one of these influences could be crystallization artifacts such as lattice contacts. Although our evaluation of the free A6 and DMF5 structures did not suggest such an influence, this along with other limitations of crystal structures still remains a potential limitation that should be considered in subsequent analyses.

### TCR binding does not induce common changes in the α or β constant domains

**Figure 5:**
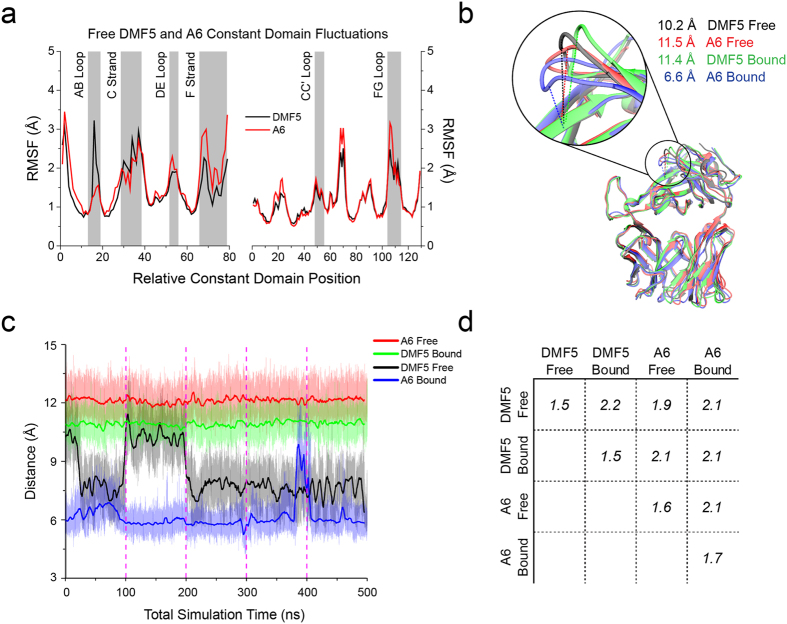
Common dynamical changes do not propagate to the A6 and DMF5 TCR constant domains upon binding. (**a**) RMS fluctuations for the A6 and DMF5 constant domains computed from 500 ns of MD simulation of the bound A6 and DMF5 TCRs. The shaded boxes indicate regions of interest. (**b**) Conformations of the AB loop within the Cα domains of the A6 and DMF5 bound and free crystal structures, showing distances between the α carbon of S127α at the loop apex and the nearby α carbon of A127β. (**c**) Distance between the α carbon of S127α of the AB loop and the α carbon of A127β in the A6 and DMF5 bound and free simulations as a function of simulation time. The loop remained static in all but two of the 20 100 ns trajectories. For free DMF5, the loop transitioned from closed to open in one trajectory (note that the loop started closed and remained so for three trajectories). Similarly, the loop transitioned from closed to open in one of the bound A6 trajectories. Transparent lines indicate frame-by-frame distance for each simulation (i.e., picosecond steps) whereas solid lines indicate the running average over 5000 frames (5 ns). Vertical dashed demark the independent 100 ns trajectories. (**d**) Matrix of average α carbon RMS deviations between the A6 and DMF5 constant domains and the bound and free simulations. Although the average conformation differs between free and bound for both A6 and DMF5 (RMSDs of 2.1 Å for A6, 2.2 Å for DMF5), conformational differences of similar magnitude exist between the free and bound states of the A6 and DMF5 constant domains (RMSDs of 1.9 Å and 2.1 Å).

We next examined the constant domains of the DMF5 and A6 TCRs in order to investigate any additional motional differences that may exist between the two TCRs. Examined by RMS fluctuations, the overall constant domain motions were generally similar, with the most pronounced difference seen in the AB loop of the distal end of the Cα domain (Fig. 5a). Different behaviors for the AB loop are of interest, as AB loop conformational changes occurring upon ligation have been proposed as a potential mechanism for TCR triggering^26^. As the AB loop showed different motional properties in the simulations of free DMF5 and A6, we asked how the constant domains and this loop in particular behaved in TCR-pMHC ternary complexes by performing 500 ns of molecular dynamics simulations for the A6 and DMF5 TCRs bound to peptide/MHC, using the same multi-trajectory methodology. For A6, we simulated the TCR bound to the Tax 11–19 peptide presented by HLA-A2. For DMF5, we simulated the TCR bound to the MART-1 26–35 ELAGIGILTV decamer bound to HLA-A2.

We first examined the conformation of the AB loop by monitoring the distance between the α carbon of S127α at the loop apex and the α carbon of the nearby A127β and comparing the measured distances to their crystallographic distances (Fig. 5b). In the free simulations, while the A6 AB loop remained in its crystallographic open conformation, in one trajectory the DMF5 AB loop transitioned into the closed conformation but did not re-open. We therefore attribute the difference in RMS fluctuations of the AB loop for the unligated TCRs to a rare conformational change resulting from a high energy barrier that was stochastically crossed in the DMF5 but not the A6 simulation. Similarly, the AB loop remained in its crystallographic open conformation in the simulation of bound DMF5, whereas in one trajectory of bound A6 the loop transitioned from its crystallographic closed conformation to an open conformation (Fig. 5c). As there was a rare conformational change in the AB loop regardless whether the TCR was free or bound, the simulations do not provide support for TCR binding altering the conformational properties of the AB loop, at least on the nanosecond timescale sampled during our simulations. This result is consistent with mutational studies indicating no impact on CD3γε binding when the AB loop is mutated^27^.

We next asked if there were motional changes elsewhere in the TCR constant domains attributable to ligand binding. For the bound and free simulations of both TCRs, we computed α carbon RMS deviations between snapshots taken every nanosecond, yielding a matrix of RMS deviations. A domain-level version of this analysis is shown in Fig. 5d. Comparing their bound and free constant domains, DMF5 and A6 differed by an average of 2.2 Å and 2.1 Å, respectively. This indicates that both sets of constant domains underwent altered conformational sampling in the bound vs. free simulations, consistent with experimental hydrogen/deuterium exchange data^28^. However, the constant domains of A6 and DMF5 in their bound states differed by a similar amount (average RMSD of 2.1 Å). Thus, although for both A6 and DMF5 the conformational sampling in the constant domains differed when the TCR was bound to pMHC, the conformations that were sampled differed between the two TCRs. As seen with the AB loop, the simulations therefore do not indicate that common alterations to constant domains are induced upon ligand binding by A6 and DMF5.

**Figure 6:**
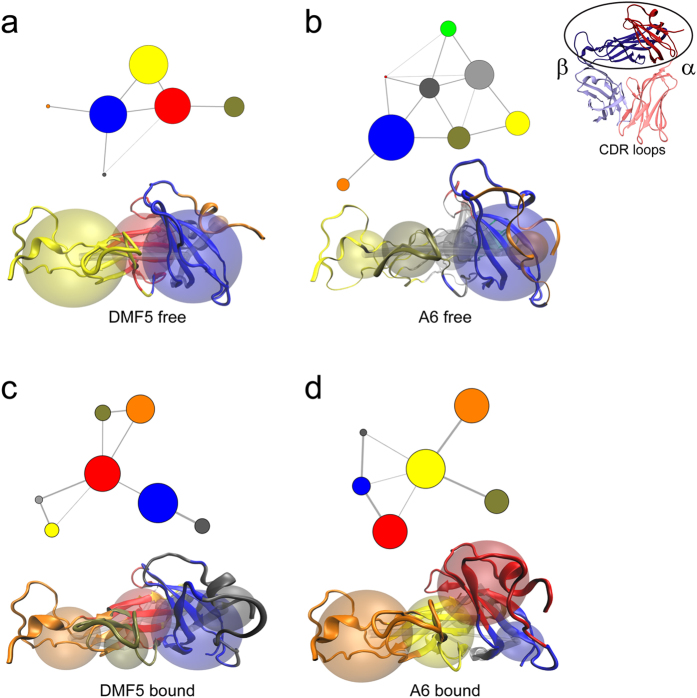
Community network analysis fails to identify common dynamical changes in the A6 and DMF5 TCR after ligand binding. (**a,b**) Community analysis for the constant domains of free DMF5 (**a**) and A6 (**b**). Communities and their connections are shown in the top images, and mapped to the three-dimensional structure of the constant domains in the bottom images. (**c,d**) Community analysis for the constant domains of bound DMF5 (**c**) and A6 (**d**). There is no apparent trend in the community assignment upon ligation, as DMF5 increased in the number of assigned communities and A6 decreased in the number of assigned communities. While communities of both TCRs were connected to the interior A, B, and E strands of the Cβ domain while bound (DMF5 red, A6 yellow), there was no impact on the correlation of α chain DE and β chain CC’ loops.

As we did not detect any common TCR structural alterations upon binding, we asked if common changes in correlated motions could be identified instead. We calculated linear mutual information for every α carbon within the constant domains, followed by Girvan-Newman clustering of the resulting matrix^29^,^30^. Similar to cross-correlation matrices, this approach reports on correlated fluctuations, but also clusters residues into “communities” of like dynamics and describes how these are linked, illustrated by the number and width of connecting edges (Fig. 6). The constant domains of the two free TCRs yielded different, non-overlapping numbers and sizes of communities (six for DMF5, eight for A6, with different connectivities). (Fig. 6a,b). The correlated motions of the constant domains of two free TCRs therefore share little in common.

The community analysis of the constant domains also did not show evidence for conserved changes upon binding. The constant domain of DMF5 slightly increased in complexity, described by seven instead of six communities. The constant domain of A6 decreased in complexity, described by six instead of eight communities (Fig. 6c,d). As for community connectivity, DMF5 changed little upon binding, whereas A6 became more connected, with each community linked via the interior A, B, and E strands of the β chain constant domain (Fig. 6d, yellow community). There were however no changes in the correlations between the α chain DE and β chain CC’ loops, regions which are believed to interact with the CD3δε and CD3γε heterodimers^27^,^31^.

### Residual motion in the TCR-pMHC interfaces and the mechanisms of cross-reactivity

Recent NMR and molecular dynamics studies of TCR-pMHC interfaces suggested motion within the complex could be related to TCR cross-reactivity and the mechanisms of peptide selectivity^18^,^32^. We thus used the simulations of the A6 and DMF5 TCR-pMHC complexes to study the two receptor-ligand interfaces. RMS fluctuations revealed that the CDR loops of both TCRs rigidified upon ligation, most notably CDR3α of both TCRs and CDR3β of A6. Small exceptions were found in the CDR1α and CDR3β loops of DMF5, which showed a slight increase in RMS fluctuations. However, this had little impact on the overall conformation of the loops, as the average RMS deviation for the loops relative to their crystallographic coordinates were 0.76 Å and 0.40 Å respectively.

The HLA-A2 α1/α2 helices in the two complexes were also relatively stable, with Cα RMS fluctuations ranging from 0.5 Å to 1.7 Å. Structural variations in the binding groove were seen in the simulation with the DMF5 complex, in which the helices deviated from their crystallographic positions to facilitate a slight opening of the groove, reflecting the variations seen in our recent analysis of TCR-pMHC crystal structures^33^. Using the distance between Tyr59 on the α1 helix Arg170 on the α2 helix to measure the groove width, we found an average distance of 17 Å for the open state in the simulation of the DMF5 complex and 14 Å in the simulation of the A6 complex. For comparison, the distances in the two crystallographic structures are 13 Å for both DMF5 and A6.

**Figure 7:**
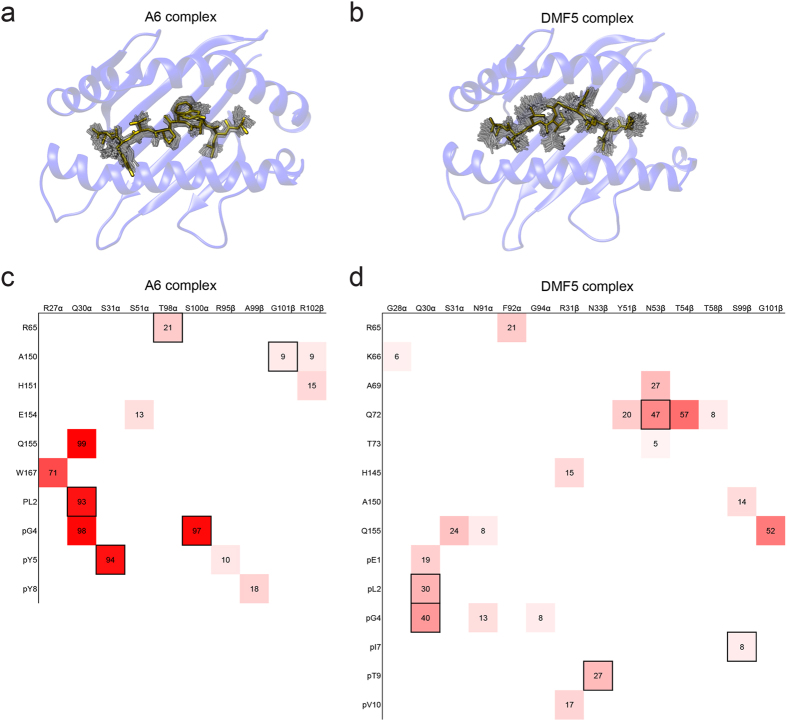
Fluctuations within the TCR-pMHC interface. (**a**) Motion of the Tax peptide in the A6 complex. The solid peptide shows the crystallographic peptide conformation, while the shaded renderings show positions extracted every nanosecond of the simulations. (**b**) As in panel **a**, but for the MART-1 peptide in the DMF5 interface. (**c**) Heat map of hydrogen bonds within the A6 interface that form during the TCR-pMHC simulation. The map shows hydrogen bonds between residues, colored by percent hydrogen bond duration during the simulation (solid red = 100%). Numbers indicate percent duration. Boxed entries show those hydrogen bonds present in the TCR-pMHC crystallographic structure. (**d**) As in panel **c**, but for the DMF5 interface.

While the Tax peptide in the simulation of the A6 complex was relatively rigid (Fig. 7a), the longer MART-1 peptide in the DMF5 complex fluctuated significantly, with the α carbons of the central isoleucine/glycine/isoleucine motif at p5-p7 fluctuating between 1.3 and 2.1 Å (Fig. 7b). While more motion is expected of the longer MART-1 peptide, the retention of peptide motion in the complex is attributable to the architecture of the binding site, in which the central “bulge” of the peptide centered at isoleucine 5 is accommodated by an open configuration of the CDR1α and CDR3α loops^13^.

Given the variations seen within the two TCR-pMHC interfaces, we investigated the stability of intermolecular contacts across the two TCR-pMHC interfaces, focusing on hydrogen bonds. A greater number of longer lasting hydrogen bonds were observed in the A6 interface (13 hydrogen bonds, with an average occupancy of 50%), particularly to the N-terminal half and central residues of the peptide (Fig. 7c). In contrast, while DMF5 formed more hydrogen bonds than A6, they were almost exclusively shorter lasting, with only two persisting longer than 50% of the total simulation time (21 hydrogen bonds, with an average occupancy of 22%) (Fig. 7d). These differences are consistent with the more open and permissive architecture of the DMF5 interface as indicated above, and ultimately the two different mechanisms of TCR cross-reactivity.

## Discussion

Discussions of how TCR CDR loop motion influences TCR recognition date back to some of the earliest TCR structural and biophysical studies. However, while structural, thermodynamic, and kinetic data can give insight into conformational changes and their relationship to motion^3^, there have been few investigations into the motional properties of TCR CDR loops and their relation to receptor binding properties. Although some progress has been made using fluorescence and NMR^18^,^20^,^25^,^34^, due to the technical challenges presented by recombinant TCRs and TCR-pMHC complexes (e.g., production yields, protein stability, binding affinities, etc.), insight in the near future is likely to emerge from computational studies^35^.

The various free and bound crystallographic structures of A6 and DMF5 suggest different mechanisms for how the two receptors bind and cross-react between different ligands. This was borne out by the studies here: unlike the A6 TCR, whose hypervariable loops move rapidly on the nanosecond timescale, the DMF5 TCR maintains a relatively rigid architecture. Accordingly, A6 relies on a rapid conformational selection mechanism in making significant adjustments for different ligands. DMF5, on the other hand, utilizes slower motions or induced-fit to make smaller adjustments to a more open and permissive binding site. As with many other features of TCRs (e.g., binding geometries and binding thermodynamics)^36^, there thus appears to be a spectrum in how motion facilitates ligand selection, binding, and cross-reactivity. This is highlighted by the striking difference in the dynamics of the A6 and DMF5 CDR3β loops: in A6 CDR3β is the most flexible of the six loops, whereas in DMF5 it is the most rigid. Simplifying generalizations about the roles of nanosecond-scale CDR loop flexibility therefore cannot easily be applied to models of TCR molecular recognition, particularly with ligand selection and cross-reactivity.

No single feature quantitatively correlated with CDR loop motion. Qualitatively, for the hypervariable loops at least, length mattered: the long hypervariable CDR3β loop of A6 was the most flexible. Beyond this though, there was no obvious relationship between sequence and structural properties and loop motion, including the presence or absence of glycines or inter-connecting hydrogen bonds. Binding site accessibility mattered, as solvent accessible surface area or atomic packing density accounted for ~60% of the motional amplitude in both TCRs. When combined with crystallographic B-factors the correlation increased to nearly 70%. Other features not easily assessed via sequence or structure likely account for the remainder, such as the differing strengths of individual inter-loop hydrogen bonds.

Interestingly, in addition to the hypervariable loops, the differences between A6 and DMF5 are due in part to differential behavior of the germline CDR1α and CDR2α loops, which despite being identical between the two TCRs, undergo slightly higher amplitude fluctuations in A6. Although the differences are small compared to the hypervariable loops, this difference highlights the inter-connected nature of TCR binding sites and how differential gene pairing and hypervariable loop sequences can have “knock on” effects not limited to static structural consequences^37^,^38^,^39^.

The binding sites of both TCRs rigidified upon binding, although as seen experimentally^18^ and in other simulations of TCR-pMHC complexes^32^,^40^,^41^,^42^, movement was still observed across the interface in both complexes. This movement mirrored the mechanisms of cross-reactivity: the architecturally more permissive DMF5 TCR formed a more dynamic interface, whereas the interface with A6 was more stable. In both cases, multiple crystallographically-observed hydrogen bonds were maintained during the simulation, reflecting the importance of hot spots in TCR-pMHC interface^23^, although again consistent with the mechanisms of cross-reactivity, greater numbers were seen in the A6 than the DMF5 interface.

Motional changes upon binding propagated to the TCR constant domains, but we were unable to identify any structural or dynamic changes common to both TCRs that could clearly be associated with TCR triggering or altered interactions with CD3 signaling modules. Although this could be used to argue in favor of TCR triggering mechanisms that depend upon ultrastructural changes of membrane-bound assemblies^8^,^43^, we caution that any molecular dynamics simulation is ultimately limited by simulation time. Our simulations addressed the nanosecond timescale, and detecting biologically-relevant binding-linked changes in TCR constant domain dynamics could require much longer simulations^44^, possibly with the membrane environment and associated signaling modules included^45^.

## Methods

### Molecular dynamics simulations

Starting coordinates for the unligated and ligated DMF5 and A6 were obtained from crystallographic structures of free TCRs or TCR-pMHC complexes deposited in the Protein Data Bank (PDB accession codes 3QEU, 3QDG, 3QH3 and 1QRN)^6^,^13^,^20^. When multiple molecules were present in asymmetric units, with the exception of unligated DMF5, starting coordinates were obtained from the first molecule. Starting coordinates for the unligated DMF5 TCR were obtained from the second molecule as it showed better disulfide bond geometry. The complex between the A6 TCR with the Tax/HLA-A2 ligand, 1QRN was used instead of 1AO7 due to its higher resolution; in this case, Ala6 of the peptide was converted to the native proline prior to preproduction. In the case of the DMF5 reverse simulation (free TCR with bound coordinates), the coordinates of 3QDG were used with the pMHC stripped off prior to preproduction procedures.

All structures were charge neutralized with explicit Na^+^ ions and subsequently solvated with explicit SPC/E waters in a cubic box using tleap from the AMBER molecular dynamics suite^46^. All systems were simulated with the GPU-accelerated code of AMBER 12^47^,^48^, using the ff99SB force field with a 2 fs time step^49^, utilized the SHAKE algorithm to restrain bonds involving hydrogen^50^, and incorporated a 10 Å cutoff for nonbonded interactions. Following generation of the initial systems, systems were energy minimized and heated to 300 K with solute restraints of 25 kcal/mol/Å^2^ in the NPT ensemble. Solute restraints were then gradually relaxed over 100 ps of molecular dynamics and the average volume of the systems with no solute restraints used as the volume to fix the systems to proceed to simulations in the NVT ensemble. Following a brief 100 ps run in the NVT ensemble velocities were scaled to 300 K and temperature restraints removed to proceed in the NVE ensemble. Following a 2 ns equilibration simulation the velocities were rescaled to 300 K and production trajectories begun. During production, coordinates files were saved every picosecond. Production trajectories were initially completed to 100 ns. For the simulations of free and complexed A6 and DMF5, the restart files at 5 ns, 10 ns, 15 ns and 20 ns were then perturbed by subjecting them to the no-restraint NPT and NVT pre-production procedures described above and subsequently re-equilibrated in order to perform four additional statistically independent 100 ns production trajectories, yielding 500 ns of simulation time for each. For the simulation of free DMF5 starting in its bound configuration, the initial 100 ns simulation was extended to 500 ns.

### Simulation and structural analyses

Trajectory analyses were performed with cpptraj from the AMBER suite^51^. Prior to analysis, trajectory coordinates were first globally superimposed to their initial crystallographic coordinates. In the case of the ligated trajectories, trajectories were superimposed onto the crystallographic coordinates of either the TCR or pMHC depending on which protein was being observed. Distances, RMS fluctuations, B-factors, RMS deviations and dynamical cross correlation matrices were calculated for the α carbons unless otherwise noted. Order parameters were calculated via isotropic reorientational eigenmode dynamic analysis using vectors defined from the Cα to Cβ (or Cα to H for glycine) atoms^52^. Hydrogen bonds were calculated using a 3.5 Å cutoff between donor and acceptor atoms and a minimum 120° A-H-B angle.

Community network analysis was performed with Bio3D using an adapted protocol from Taylor and colleagues^29^,^53^. Briefly, mutual information was calculated for the dynamics of each α carbon within the TCR constant domains. The resulting matrix was subjected to a Girvan-Newman clustering protocol in order to cluster the dynamics of the constant domain into communities of like dynamics^30^. Prior to clustering, edges were removed for mutual information values <0.4 and pairs of atoms whose average simulation distance was greater than 8–10 Å, chosen as the value which yielded the smallest number of community assignments. In the case of an equal number of communities for different cutoff values, the larger cutoff value was used.

Residue-specific solvent accessible surface areas, normalized to values from Ala-X-Ala tripeptides, were calculated with Accelrys Discovery Studio using a using 1.4 Å radius probe and 240 grid points per atom. Atomic packing densities were calculated from Voronoi volumes using the Voronoia webserver (http://bioinformatics.charite.de/voronoia)^54^. Multiple linear regression was performed with Origin 9, fitting to RMSF = *a*(relative SASA) +*b*(B-factor) +*c*. Images were rendered in either VMD, Chimera, or Discovery Studio^55^,^56^. Data was plotted with Origin 9.

### Structure and charge derivation of cysteine covalently linked to fluoroscein-5-maleimide

A geometry optimized structure of cysteine covalently linked to fluoroscein- 5-maleimide for use in simulation was prepared in Gaussian09 utilizing the HF/631-G(d) basis set. Topology and force field parameters for the structure were generated by Antechamber, a set of tools included in the AMBER suite. The structure was then inserted into two sites on the DMF5 TCR: R27α (CDR1α) and F100β (CDR3β), each mutated to cysteine for probe conjugation. Pre-production and production trajectories of the new structures were performed as described above.

### DNA mutagenesis, recombinant protein expression, and purification

Genes for the ectodomains of the DMF5 α and β domains were previously inserted into the pGMT7 vector^13^. Cysteine mutants were generated via QuickChange mutagenesis. Following sequence verification, the DNA constructs for the wild type and mutant α and β chains were transformed and grown in BL21 *E. Coli*. Following growth, the cells were lysed and inclusion bodies purified according to standard procedures^57^. Wild-type and mutant DMF5 TCRs were refolded by dilution and dialysis as described previously^13^,^58^. Refolded protein was purified by ion exchange followed by size exclusion chromatography.

### TCR labeling and fluorescence anisotropy

Protein labeling and fluorescence anisotropy was performed as described previously^25^,^33^. Briefly, approximately 25–50 μM of purified wild type and cysteine mutant DMF5 TCR was combined with a ten-fold excess of fluorescein-5-maleimide and 20uM TCEP-HCl. The reaction was allowed to mix at room temperature for 75 minutes and subsequently dialyzed for 18 hours and re-purified via size exclusion chromatography to remove free fluorescein-5-maleimide. To verify correct labeling, all three samples were run on reduced and nonreduced SDS-PAGE gels, which were then imaged with UV light and stained with Coomassie blue. Labeling efficiency, determined via the ratio of absorbance at 492 nm and 280 nm, was 0% for the wild type TCR and >50% for both mutants. Steady state fluorescence anisotropy experiments were performed on a Beacon 2000 instrument. Measurements were performed at 10 °C and at protein concentrations of 75 nM and 150 nM. 50 measurements were performed for each sample after they were allowed to reach thermal equilibrium.

## Additional Information

**How to cite this article**: Ayres, C. M. *et al.* Differential utilization of binding loop flexibility in T cell receptor ligand selection and cross-reactivity. *Sci. Rep.* **6**, 25070; doi: 10.1038/srep25070 (2016).

## Figures and Tables

**Table 1:** A6 and DMF5 CDR loop sequences.

	DMF5	A6
CDR1α	DRGSQS	DRGSQS
CDR2α	IYSNGD	IYSNGD
CDR3α	AVNFGGGKLI	AVTTDSWGKLQ
CDR1β	MRHNA	MNHEY
CDR2β	SNTAGT	SVGAGI
CDR3β	ASSLSFGTEAF	ASRPGLAGGRPEQY
